# Heavy Metal(loid)s Contamination in Ground Dust and Associated Health Risks at a Former Indigenous Zinc Smelting Area

**DOI:** 10.3390/ijerph18030893

**Published:** 2021-01-21

**Authors:** Shan Li, Xiangyang Bi, Zhonggen Li, Heng Wang, Xinyu Li, Xinbin Feng, Guangyi Sun, Ji Chen, Bo Meng

**Affiliations:** 1 State Key Laboratory of Environmental Geochemistry, Institute of Geochemistry, Chinese Academy of Sciences, Guiyang 550081, China; lishan@mail.gyig.ac.cn (S.L.); lixinyu@mail.gyig.ac.cn (X.L.); fengxinbin@mail.gyig.ac.cn (X.F.); sunguangyi@mail.gyig.ac.cn (G.S.); chenji@mail.gyig.ac.cn (J.C.); 2 University of Chinese Academy of Sciences, Beijing 100049, China; 3 State Key Laboratory of Biogeology and Environmental Geology, School of Earth Sciences, China University of Geosciences, Wuhan 430074, China; bixy@cug.edu.cn; 4 School of Resources and Environment, Zunyi Normal College, Zunyi 563006, China; 5 School of Public Management, Guizhou University of Finance and Economics, Guiyang 550025, China; hengwang198510@126.com

**Keywords:** zinc smelting activities, ground dust, contamination level, exposure risks, temporal changes

## Abstract

Indigenous zinc smelting (IZS) is a backward technique that releases a great deal of heavy metal(loid)s into the environment. However, the contamination of heavy metal(loid)s in ground dust and the associated health risks in such areas are poorly known. In this study, a former IZS area in Guizhou, China, was surveyed during 2008–2018 with 15 elements (Ag, As, Bi, Cd, Co, Cr, Cu, Hg, In, Ni, Pb, Sb, Sn, Tl, Zn) being analyzed. The results indicate that most elements (e.g., Ag, As, Cd, Cu, Pb, Sb, Sn, Zn) in ground dust decreased significantly after the cessation of the IZS in 2006; nevertheless, some elements still remained at relatively high levels in 2018, e.g., Pb (average: 762 ± 647 mg/kg), Zn (average: 1287 ± 753 mg/kg), Cd (average: 7.76 ± 5.06 mg/kg), and As (average: 41.9 ± 34.8 mg/kg), indicating they might come from the local contaminated soils, slag residues and smelting potteries. In terms of the impacts on human health, children have both higher non-carcinogenic and carcinogenic risks than that of adults, with the latter subpopulation having a lower risk than the threshold values. Pb and As were the two elements with the highest non-carcinogenic risk for children, the hazard index of local children was still higher than the threshold of 1 (e.g., 1.43 for As, 2.09 for Pb) in 2018. The carcinogenic risk of As exposure to children dropped more than two times to 6.42 × 10^−7^ in 2018, which falls below the tolerable range (10^−6^–10^−4^). This study revealed that although the concentration of heavy metal(loid)s in ground dust and linked health risk in the IZS area has reduced dramatically after the cessation of IZS, continued removal of slag residues and smelting potteries is necessary for further decreasing the human health risk.

## 1. Introduction

Heavy metal(loid)s could induce a serial of adverse effects on human beings and animals and deteriorate the environment through natural and anthropogenic activities [1,2,3,4]. Lead-zinc smelting is an important source of potentially toxic elements [5,6,7]. China ranked the largest Zn/Pb/Cu producer in the world for more than 20 years [8]. Besides the large-scale smelting technique, small-scale or indigenous smelting had played an important role in the past decades in some areas of China, such as Sichuan, Yunnan, and Guizhou provinces in Southwest China [8,9,10]. Indigenous Zn smelting (IZS) in Northwestern Guizhou Province is more notable for its bigger scale and duration than other provinces due to the abundance of zinc and coal resources in this area and the increasing domestic demands [11]. The local IZS last for several centuries and the number of the IZS sites peaked in the 1980s and 1990s, when an output of Zn ingot reached more than 100 kt/yr [12]. Specially, a simple pyrometallurgy technique was employed during the IZS activities, in which coal was used as a fuel and reducing agent with poor pollution control measures. Consequently, the recovery of zinc resources during the IZS process was low (50–60%, [9]) but with relatively higher atmospheric emission factors for other metal(loid)s, such as Pb, Cd, Hg, and As [10,13,14]. The average Hg emission factors are 155 and 79 g Hg per ton of Zn produced from sulfide and oxide ores, with Cd emission factors reaching up to 1460 and 1240 g Cd t^−1^, respectively [11,15]. In addition, massive sterile wastes and tailings (2.1 × 10^7^ t) were produced and discarded locally during the IZS activities, which occupied 1200 hm^2^ of area [16]. More importantly, high levels of toxic metals in the discarded slags were readily transported into the environment through natural weathering and other processes under low pH conditions [17,18]. Previous observation showed that a large amount wastewater containing 2.23 tons of Pb and 61 kg of Cd have resulted in serious Pb/Cd contamination to local stream rivers in Northwestern Guizhou, China [19]. The death of fish and shrimp within dozens of kilometers from the IZS site have been attributed to the Pb/Cd poisoning of heavy metal-contaminated water [19]. Local vegetables, crops, and even the traditional Chinese medical plants were also toxic metal(loid)s-contaminated in the IZS areas, hence, this activity contaminated the environment and posed a potential threat to residents [10,20].

**Figure 1:**
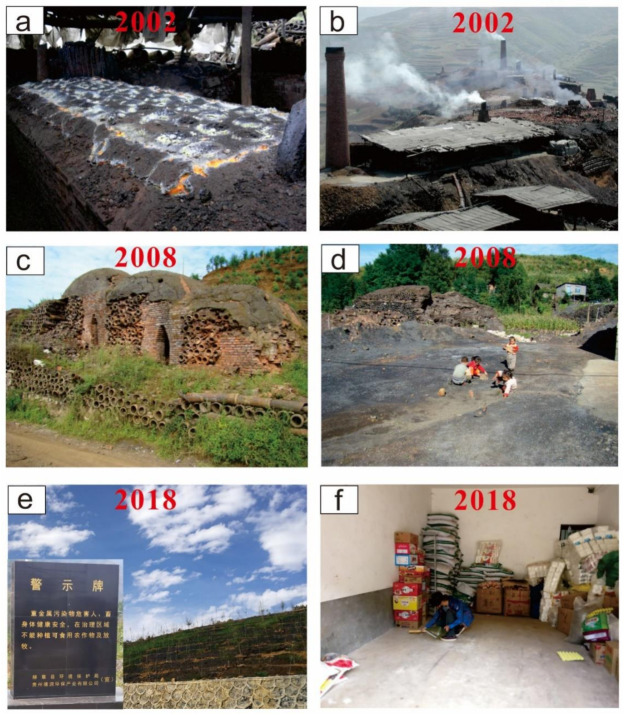
Dynamics of the indigenous zinc smelting (IZS) in Hezhang, Guizhou. (**a** and **b**, IZS activities in Hezhang before 2006; **c** and **d**, closed IZS furnace and slag piles in 2008 two years after the complete ceasing of IZS; **e**, Disposed zinc smelting slags after the 2010s; and **f**, ground dust collection in residential houses in 2018).

**Figure 2:**
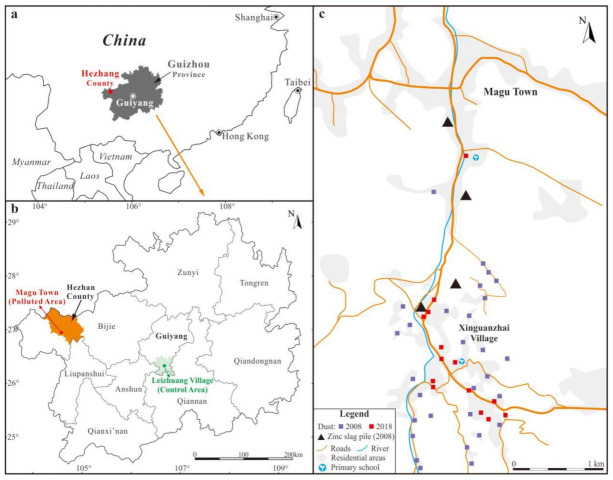
Relative location of the study areas in (**a**) China and (**b**) Guizhou, and (**c**) the detailed sampling sites in the IZS area.

**Figure 3:**
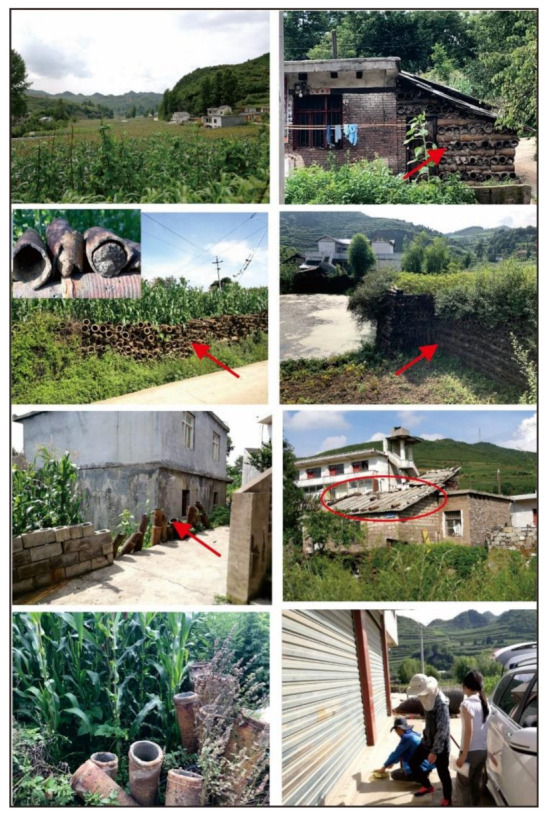
Legacy smelting pottery in Xin-Guan-Zhai village in 2018 and the on-site dust sampling.

The IZS involved a lot of villagers from 1980s to 2000s since it was a high-income business at that time and the smelting sites were often close to or inside the villages (Figure 1 and Figure 2), where the smelting furnaces were set up, where coal and zinc concentrate from the same county was transported to, and where zinc ingots were produced and the smelting slags were produced and thereby discarded (Figure 1). After the complete banning of IZS in 2006, the vast majority of smelting slag piles had not been treated for the first several years until 2010s when they were disposed of or landfilled by the local authorities to isolate these hazardous materials from the surface ecosystem (Figure 1). However, the smelting tool for holding the zinc concentrate (mortars) during smelting was still widely distributed in the village in the following years, being used for fences, walls, or as a weight (Figure 3).

To date, the heavy metal contamination in ground dust of these smelting villages or areas and the associated health risks in the IZS areas have not drawn much attention, and there is little information available concerning these two important aspects. Ground dust is a comprehensive indicator of pollution levels and is believed to present a higher pollution level in mining/smelting areas; thus, the effects of heavy metals in ground dust on human health cannot be ignored. The consensus is that the ingestion of dust particles appears to be the main route of heavy metal exposure to residents. More importantly, children are more vulnerable because of hand-to-mouth behavior, proximity to the floor, and different metabolisms when compared to adults [21,22,23].

Although the IZS activities in Northwestern Guizhou Province were terminated in 2006, high levels of Pb (mean ± standard deviation (SD): 3510 ± 4690 mg kg^−1^, range: 6–19,400 mg kg^−1^), Zn (mean ± SD: 6520 ± 8260 mg kg^−1^, range: 5–36,500 mg kg^−1^), Cd (mean ± SD:13.3 ± 12.1 mg kg^−1^, range: 0.04–53.7 mg kg^−1^), and As (mean ± SD: 169 ± 290 mg kg^−1^, range: 3–1320 mg kg^−1^) in soil were observed by other researchers 10 years after the cessation of IZS activities [24]. As an important environmental media, ground dust has not been studied and reported for the heavy metal contamination, and the impact on human health is unclear. To fill these research gaps, a typical former IZS area located in Hezhang county, Northwestern Guizhou Province, China was selected; Guiyang city (the capital of Guizhou Province) was chosen as a control site. Ground dust samples both from IZS site and control site were collected in 2008 and 2018 for heavy metals analysis. The primary objectives of this study are (1) to investigate the long-term temporal variations of heavy metal(loid)s in ground dust of the IZS area, and (2) to evaluate the contamination status of these elements in the IZS area and related health risks to the local residents who were co-exposed to multiple heavy metal(loid)s. The results of this study will provide environmental managers with critical information for assessing the local environmental quality and taking necessary actions to further remediate the contamination in ground dust in these areas.

## 2. Materials and Methods

### 2.1. Study Area and Sample Collection

The historical IZS activities in Magu town of Hezhang county can be dated back to 10th century (947 AD), and the IZS activities appeared in its heyday during the period from 1980s to early 2000s (Figure 1a,b), with more than 520 “Macao local small furnaces” distributed in this area [12]. Magu town was known in China for its contribution to the environmental contamination/destruction. Xin-Guan-Zhai village is recognized as a typical IZS area close to Magu town (<5 km); the abandoned furnaces and large quantities of smelting slag were deposited along the village road after the secession of IZS in 2006 (Figure 1c,d). The IZS activities in Xin-Guan-Zhai village have resulted in serious heavy metal contamination (e.g., Cd, Zn, Pb) to ambient soils, sediments, and agricultural crops [25] through the discharge of flue gas and slags (Figure 1a,b).

In this study, Xin-Guan-Zhai village was selected as the zinc smelting impacted area (Figure 1 and Figure 2) and Leizhuang village in suburb Guiyang was selected as the control site, which experiences a similar climate in comparison to the Xin-Guan-Zhai village (IZS area) but has no IZS or other direct heavy metal point contamination sources. The control site of Leizhuang is located in the southwest of Guiyang city, the capital of Guizhou province, ~35 km away from the city center and ~300 km away from the IZS area. Magu town experiences a subtropical monsoon climate with an average annual rainfall of 900 mm and an annual average temperature of 12.5 °C. The total area of Magu town is 141.93 km^2^, with a lowest and highest elevation of 1775 m and 2588 m, respectively. Corn is the predominant agricultural crop in the study area, which accounts for 45–55% of the total annual production, followed by rice, potatoes, wheat, and soybeans.

In this study, thirty-seven and fourteen ground dust samples were randomly collected from the whole Xin-Guan-Zhai village in Sep. 2008 and July 2018, respectively, to disclose the overall contamination of this village, since the whole village was involved in the IZS, with smelting furnaces and slag piles having been concentrated in the north part of village (Figure 2). This village is located in a valley surrounded by small hills (< 200 m in height) and a small stream that flows away (Figure 2 and Figure 3). Furthermore, fifteen ground dust samples were collected from the control site of Leizhuang village in Sep. 2008 (Figure 2). No samples were collected in the control area in 2018 since there was no industrial activities and the living conditions of local residents did not change too much during the study period, hence the levels of heavy metals in ground dust were assumed to be similar to that in 2008. In detail, ground dust samples (30–100 g for each of the sampling site) were collected with a clean plastic dustpan and a brush from the village roads (mostly unpaved roads in 2008 and paved roads in 2018), residential yards (mainly paved), and living rooms within an area of 5–10 m^2^. All dust samples were collected by hand with a disposable polyethylene glove and stored in polyethylene bags to avoid cross contamination before being brought back to the laboratory and finally air-dried. The stone, glass, and cigarette butts were removed, and the samples were then ground with an agate mortar and sieved through 100 mesh (0.15 mm) nylon sieves; only the portion lesser than 0.15 mm was subjected for heavy metal(loid)s analysis.

### 2.2. Analytical Methods for Hevy Metal(loid)s

A total of 15 elements, including silver (Ag), arsenic (As), bismuth (Bi), cadmium (Cd), cobalt (Co), chromium (Cr), copper (Cu), mercury (Hg), indium (In), nickel (Ni), lead (Pb), antimony (Sb), tin (Sn), thallium (Tl), and zinc (Zn), were analyzed in the dust samples. Total Hg content in dust samples was measured using a Milestone™ direct mercury analyzer (Model DMA 80, AMA 254 Software) following USEPA method 7473 [26]. Arsenic was determined using atomic fluorescence spectrometry (AFS-920, Beijing Jitian Instrument Corporation) after the sample was digested with HNO_3_-HCl-H_2_O_2_ following USEPA method 3050B [27]. For the analysis of other thirteen elements, approximately 50 mg of sample was transferred into a 10 mL Teflon digestion tank and digested with a freshly prepared mixture of 2 mL concentrated HF/HNO_3_ (v/v, 1:1). Then, the tank was tightly capped and heated in muffle (190 °C) for 24 h. Once cooled down, the tank was uncapped and re-heated on a hot plate at 150 °C to completely evaporate the digest. The digest was then evaporated to dryness after the addition of 0.5 mL HNO_3_. Finally, 2 mL HNO_3_, 2 mL deionized water, and 500 ng of Rh (internal standard) were added into the digestion and heated for additional 5 h at 140 °C [28]. A suitable volume of aliquot from the digested sample was taken for trace heavy metal(loid)s analysis by inductively coupled plasma-mass spectrometer (ICP-MS, ELAN DRC-e, PerkinElmerSCIEX, Shelton, CT, USA).

### 2.3. QA/QC and Statistical Analysis

Quality assurance and quality control (QA/QC) for elements analysis in the dust samples was conducted using method blanks, duplicates, and standard reference materials (non-contaminated soil GSS-5 and contaminated soil NIST 2710). The recoveries of the elements ranged from 90.6% to 107.2%. The relative standard deviation for analysis of duplicate samples was less than 5%.

Statistical analysis was performed using Excel 2019 and IBM SPSS Statistics 25 for Windows. One-way analysis variance (ANOVA) and independent-samples t test in SPSS were performed to compare significant differences between two independent datasets, e.g., between different areas (zinc smelting and control areas) or different periods (2008 and 2018). Significant differences between these average values are indicated by a *p* value less than 0.05. The data were plotted using Origin Pro. 2018.

### 2.4. Pollution Assessment Method

The pollution status of a single element was assessed using the geo-accumulation index (*I_geo_*) approach [29], which was popularly employed in previous study [30,31]. *I_geo_* was calculated using equation (1):

(1) $$I_{\mathrm{geo}}{=\log}_{2}\frac{C_{n}}{{k\text{×}B}_{n}}.$$

where C_n_ is the concentration of heavy metal(loid)s in dust (mg/kg); B_n_ is the elemental geochemical background concentration (mg/kg). In this study, the soil background value in Guizhou Province was taken as a reference to eliminate the influence geological background [32]; k is a correction coefficient (typically 1.5) used to consider the variations in the background value caused by diagenesis. The standard classifications of *I_geo_* for heavy metals are divided into seven class, Class 0, *I*_geo_≤ 0, uncontaminated; Class 1, 0 < *I*_geo_ ≤ 1, uncontaminated to moderately contaminated; Class 2, 1 < *I*_geo_ ≤ 2, moderately contaminated; Class 3, 2 < *I*_geo_ ≤ 3, moderately to strongly contaminated; Class 4, 3 < *I*_geo_ ≤ 4, strongly contaminated; Class 5, 4 < *I*_geo_ ≤ 5, strongly to extremely contaminated; Class 6, *I*_geo_ ≥ 5, extremely contaminated [29].

### 2.5. Health Risk Assessment

The ground dust can impact human health through ingestion, inhalation, and dermal contact [33,34]. Therefore, there are usually two methods to assess such health risks, with one of the bioaccessibility assessments simulating lung and gastric-intestinal fluids [35,36] and one with exposure assessment models developed by the U.S. Environmental Protection Agency [37]. The latter method was used in this study, with three main routes of human exposure, including direct ingestion of substrate particles (ADD_ing_), inhalation of re-suspended particles through mouth and nose (ADD_inh_), and dermal absorption of trace elements in particles adhered to the exposed skin (ADD_dermal_), being calculated using Equations (2)–(4). For Hg, exposure via inhalation of its elemental vapor was evaluated using Equation (5). For carcinogens, the lifetime average daily dose (LADD) was used for cancer risk assessment and calculated as a weighted average for each exposure route (Equation (6)):

(2) $${\mathrm{ADD}}_{\mathrm{ing}}=\frac{{C\text{×}\mathrm{EF}\text{×}\mathrm{ED}\text{×}\mathrm{IngR}\text{×}10}^{-6}}{\mathrm{AT}\text{×}\mathrm{BW}}.$$

(3) $${\mathrm{ADD}}_{\mathrm{inh}}=\frac{C\text{×}\mathrm{EF}\text{×}\mathrm{ED}}{\mathrm{AT}\text{×}\mathrm{BW}}\text{×}\frac{\mathrm{InhR}}{\mathrm{PEF}}.$$

(4) $${\mathrm{ADD}}_{\mathrm{dermal}}=\frac{{C\text{×}\mathrm{EF}\text{×}\mathrm{ED}\text{×}\mathrm{SL}\text{×}\mathrm{SA}\text{×}\mathrm{ABS}\text{×}10}^{-6}}{\mathrm{AT}\text{×}\mathrm{BW}}.$$

(5) $${\mathrm{ADD}}_{\mathrm{vapour}}=\frac{C\text{×}\mathrm{InhR}\text{×}\mathrm{EF}\text{×}\mathrm{ED}}{\mathrm{VF}\text{×}\mathrm{BW}\text{×}\mathrm{AT}}.$$

(6) $$\mathrm{LADD}=\frac{C\text{×}\mathrm{EF}\text{×}\mathrm{ED}\text{×}\mathrm{CR}}{\mathrm{AT}\text{×}\mathrm{BW}\text{×}\mathrm{PEF}}.$$

where C is the concentration of heavy metal (loid) in the ground dust; EF is the exposure frequency; ED is exposure duration; IngR and InhR is the ingestion and inhalation rate, respectively; AT is the averaging time; BW is the average body weight; PEF is particle emission factor; SL is the skin adherence factor; SA is the exposed skin area; ABS is dermal absorption factor (unitless); VF is the volatilization factor; CR is contact (absorption) rate. The details of these parameters are listed in Table S1 in the supporting information. Considering that the concentration of each trace element followed the log-normal distribution, the 95% upper confidence limit (UCL) was calculated using the statistical software SPSS.

The potential non-carcinogenic and carcinogenic risks for humans were calculated using Equations (7) and (8) [38].

(7) $$\mathrm{HI}=\sum {\mathrm{HQ}}_{i}=\sum \frac{{\mathrm{ADD}}_{\mathrm{ij}}}{{\mathrm{RfD}}_{\mathrm{ij}}}.$$

(8) $$R_{i}{=\mathrm{LADD}}_{\mathrm{ij}}{\text{×}\mathrm{SF}}_{\mathrm{ij}}.$$

where HI is the hazard index and represents total non-carcinogenic risk, which equals to the sum of the HQ values; HQ_i_ is the risk associated with non-carcinogenic heavy metal i; ADD_ij_ is the average daily exposure to non-carcinogenic heavy metal i through j exposure routes(mg·kg^−1^·day^−1^); RfD_ij_ is the reference dose (mg·kg^−1^·day^−1^); R_i_ is the risk associated with carcinogenic heavy metal i; LADD_ij_ is the average end-life exposure to carcinogenic heavy metal i through j exposure routes(mg·kg^−1^·day^−1^); SF_ij_ is the slope factor of carcinogenic heavy metal i (kg·day·mg^−^^1^). Reference dose and carcinogenic slope factor values for different exposure routes of heavy metal(loid)s are shown in Table S2. If the value of HI is less than one, it is assumed that there is no significant risk of non-carcinogenic effects. If HI exceeds one, then there is a chance that non-carcinogenic effects occur, with a probability which tends to increase as the value of HI increases [37]. If carcinogenic risk < 1 × 10^−6^, the carcinogenic risk is negligible; if carcinogenic risk > 1 × 10^−4^, the risk of developing cancer becomes high; and if carcinogenic risk values remain within the range of 1 × 10^−6^ and 1 × 10^−4^, it is an acceptable or tolerable risk to social stability and human health [38].

## 3. Results and Discussion

### 3.1. Concentrations of Heavy Metal(loid)s in Ground Dust

Concentration of heavy metal(loid)s in ground dust collected from the IZS area and control sites are shown in Table 1; the large variation of most elements, especially in the IZS area, indicates a high skewness of data. The mean concentrations of different elements in ground dust from the IZS area collected in 2008 increased in the order of Hg, In, Tl, Ag, Bi, Sb, Sn, Co, Cd, Ni, Cr, As, Cu, Pb, and Zn, and with the order of Hg, Tl, In, Ag, Bi, Cd, Sn, Sb, Co, As, Ni, Cr, Cu, Pb, and Zn in 2018. Although the sequence of element abundance is slightly different between 2008 and 2018, Zn and Pb were confirmed as the most abundant elements. In 2008, Zn and Pb in the dust of IZS area ranged from 256 to 8245 mg/kg and 220 to 6348 mg/kg, respectively. The average values of Zn and Pb in dust samples from IZS area were more than 38 and 70 times of Guizhou soil background (89.94 mg/kg for Zn, 29.39 mg/kg for Pb, [32]), indicating extreme enrichment. In 2018, the concentrations of Zn and Pb ranged from 448 to 2849 mg/kg and from 169 to 1974 mg/kg, respectively. The mean concentrations of Zn (1287 mg/kg) and Pb (762 mg/kg) in 2018 decreased by more than 60% compared to those in 2008, however, these values obtained after ceasing the IZS activities 12 years were still 14 and 26 times higher than the soil background values in Guizhou Province. Studies have shown that the anthropogenic enrichment of zinc and lead in urban samples has reached a medium level, indicating the contribution of traffic emissions [39]. For this study area, the excess accumulation of lead and zinc in dust is mainly attributed to industrial activities. The serious contamination of Zn and Pb in ground dust can be attributed to the improper treatment of mortars which is a pottery holding Zn concentrate and has been used as a smelting tool for IZS and now is used for fences, walls, and other uses by local people (Figure 3). In addition, the polluted local soils surrounding the dwelling houses would be another source of heavy metal(loid)s in dust [24], since plenty of activities, e.g., the soil tillage, wind erosion, and walking, could bring the contaminated soil into the ground dust [40]. Average concentrations of other metal(loid)s (Ag, As, Cd, Cu, Sb, and Sn) in ground dust decreased by 44%, 62%, 70%, 74%, 45%, and 58% at the IZS area during the period from 2008 to 2018, respectively (Table 1). Furthermore, the average concentrations of these elements in 2018 were 5.06, 2.81, 24.78, 3.29, 8.07, and 2.69 times when compared to the background soil in Guizhou Province [32], respectively. Differently, concentrations of Bi, Co, Cr, Hg, In, Ni, and Tl in the dust samples from the IZS area remained almost unchanged over the period of 2008–2018. High concentrations of heavy metal(loid)s in the dust samples of the IZS area in 2008 resulted from the waste residue piles since most of them were not properly treated (Figure 1d). In 2018, high values of Pb, Zn, and Cd were detected in locations that had mortar as fences, and the lowest values of these elements were observed in newly built houses. In contrast to the soil heavy metal pollution in the IZS area that kept relative stable over time [24], heavy metal(loid)s contamination in ground dust had been greatly mitigated via the smoke reduction after the cessation of IZS activities and the treatment of smelting residues in 2010s by blocking the source of heavy metal(loid)s.

Compared with the control area (Table 1), nine elements (Ag, As, Bi, Cd, In, Pb, Sb, Sn, Zn) in the IZS area in 2008 and 2018 had elevated concentrations, indicating the impact of IZS on the accumulation of metal(loid)s in local ground dust. No significant differences were observed between the IZS sites and the control sites for Co, Cr, Hg, Ni, and Tl concentrations, which can be explained by several reasons. Firstly, Co, Cr, and Ni are not associated with Zn ores [41]; secondly, Hg emitted in the flue gas with depletion in the residues [15]; thirdly, much lower Tl concentrations (<1 mg/kg) were observed in the local Zn ore [42] than those used in other Zn smelters in China (Tl: 15–88 mg/kg) [43]. On the contrary, Cu was remarkably higher in the control area than the IZS area, likely because of the common use of copper-containing products, devices, or wires. In addition, the concentration of Cu is related to traffic emissions [44].

The results suggested that the concentrations of over a half element (Zn, Pb, Cu, Cd, Ag, As, Sn, and Sb) in dust samples were linked to the IZS activities since the significantly decreased values in 2018 were compared to those in 2008 (Table 1). A similar trend was observed in Avilés [45], an industrial area in Spain, where some industrial activities have declined, with concentrations of Zn, Cd, Hg, Ag, and As in ground dust decreasing over time (1996–2001, 2001–2006) in four surveys (1996, 2001, 2006, 2011). Although zinc smelting has been banned for many years in the study area, its impact last a long time, as shown by the excess background values in varying degrees in the following ascending order: Tl, Sn, As, Hg, Cu, In, Sb, Bi, Ag, Cd, Zn, Pb. High concentrations of Zn, Cd, and Pb were also observed in the street dust in Huludao City that was impacted by the large-scale Zn smelting activities [22].

In this study, the concentrations of heavy metals in dust samples from the IZS area were at the same levels when compared to other cities that were influenced by large-scale Pb/Zn smelting activities, such as Zhuzhou [41] and Huludao [21] in China, and Avilés in Spain [5,45]. However, heavy metal concentrations in dust samples from the IZS area were much higher than those of other locations with an absence of non-ferrous smelting activities (Table 2). Even in 2018, the concentrations of most heavy metal(loid)s, such as As, Cd, Pb, and Zn, in ground dust at the IZS sites were approximately ten times higher than those in other non-smelting cities, indicating the ongoing severity of contamination in the study area.

### 3.2. Contamination Degree of Heavy Metal(loid)s in Ground Dust

**Figure 4:**
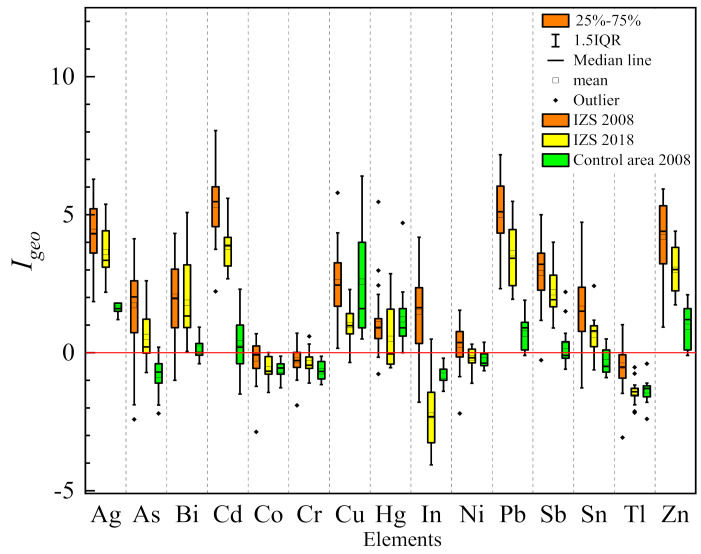
Heavy metal(loid) contamination of ground dust in this study revealed by *I*_geo_ values (Note: 1.5IQR means 1.5 times quartile spacing).

The average contamination degree of ground dust in the IZS area and control site is listed in Table S3. The comparison of the geo-accumulation index of each element in dust samples during the period of 2008 to 2018 is shown in Figure 4. The geo-accumulation index of most elements in dust from the IZS area in 2018 was lower than that in 2008. For samples collected in 2008, the average *I*_geo_ value of Co, Cr, and Tl was less than 0 (uncontaminated), with the corresponding values higher than 0 for other elements, indicating different accumulation levels between the two-group elements. Most elements had *I*_geo_ > 2: Sb (2.88), Cu (2.55), Bi (2.02) (2 < *I*_geo_ ≤ 3: moderately to heavily contaminated), or even higher than 4, such as Zn (4.19), Ag (4.38), Pb (4.99) (4 < *I*_geo_ ≤ 5: heavily to extremely contaminated), and Cd (5.36) (*I*_geo_ ≥ 5: extremely contaminated), indicating the extra Zn, Ag, Pb, and Cd accumulation in ground dust (Table S3 in Supporting Information). For the samples collected in 2018, clearly decreased *I*_geo_ values for most elements were observed, while *I*_geo_ values of Zn (3.00), Pb (3.60), Ag (3.64), and Cd (3.83) remained relatively higher (3 < *I*_geo_ ≤ 4: heavily contaminated). This is compared with the control area where the contamination levels of different elements were very low, with all *I*_geo_ values lower than 1 (0 < *I*_geo_ ≤ 1, uncontaminated to moderately contaminated), and some less than 0 (uncontaminated).

### 3.3. Human Health Risks of Heavy Metal(loid)s in Ground Dust

#### 3.3.1. Non-Carcinogenic Risk to Residents

**Figure 5:**
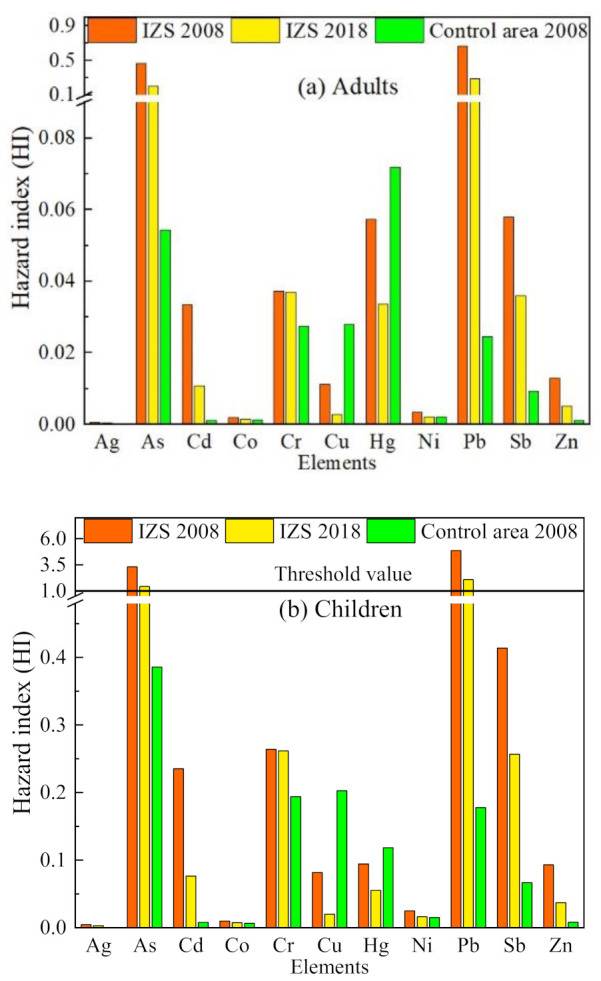
Hazard index (HI) of heavy metal(loid)s in ground dust to adults (**a**) and children (**b**).

The hazard index (HI) of non-carcinogenic risk of metal(loid)s in ground dust to residents is shown in Figure 5. The value of HI <1 indicates a negligible non-carcinogenic risk to residents, whereas when HI >1 there is a concern for non-carcinogenic health effects [37]. For adults, there was no risk of multiway exposure in both the IZS area and the control site (Figure 5a), whereas the situation for children was not optimistic (Figure 5b). In 2008, the HI values of As and Pb for children were 3.31 and 4.86, respectively (Table S4), with values far exceeding 1. Despite the significantly decreasing heavy metals concentration in dust samples, the HI values of As and Pb in 2018 were still over the acceptable value (1.43 for As, 2.09 for Pb) (Table S5), indicating that As and Pb posed non-carcinogenic health risks to children even a decade after the termination of IZS. Furthermore, the HI of children was 2–7 fold higher than that of the adults in most cases (Tables S4–S6), suggesting that children were more susceptible to similar exposure levels. This is due to the fact that children have frequent hand-to-mouth behavior, lower body weight, closer proximity to the floor and different metabolisms compared to adults. The contribution of different exposure routes was ranked as follows: ingestion > dermal contact > inhalation for both adults and children (Tables S4–S6). This sequence applies to all elements in this study with one exception (Hg); the inhalation of Hg vapor was the principal exposure route instead of particulate matter, which agrees with the previous study [54]. The results demonstrated that As and Pb were the most important pollutants in ground dust of the IZS area that posed a non-carcinogenic health risk to residents, especially children. Thus, the potential health risks to children resulting from exposure to ground dust cannot be neglected.

#### 3.3.2. Carcinogenic Risk to Residents’ Health

**Figure 6:**
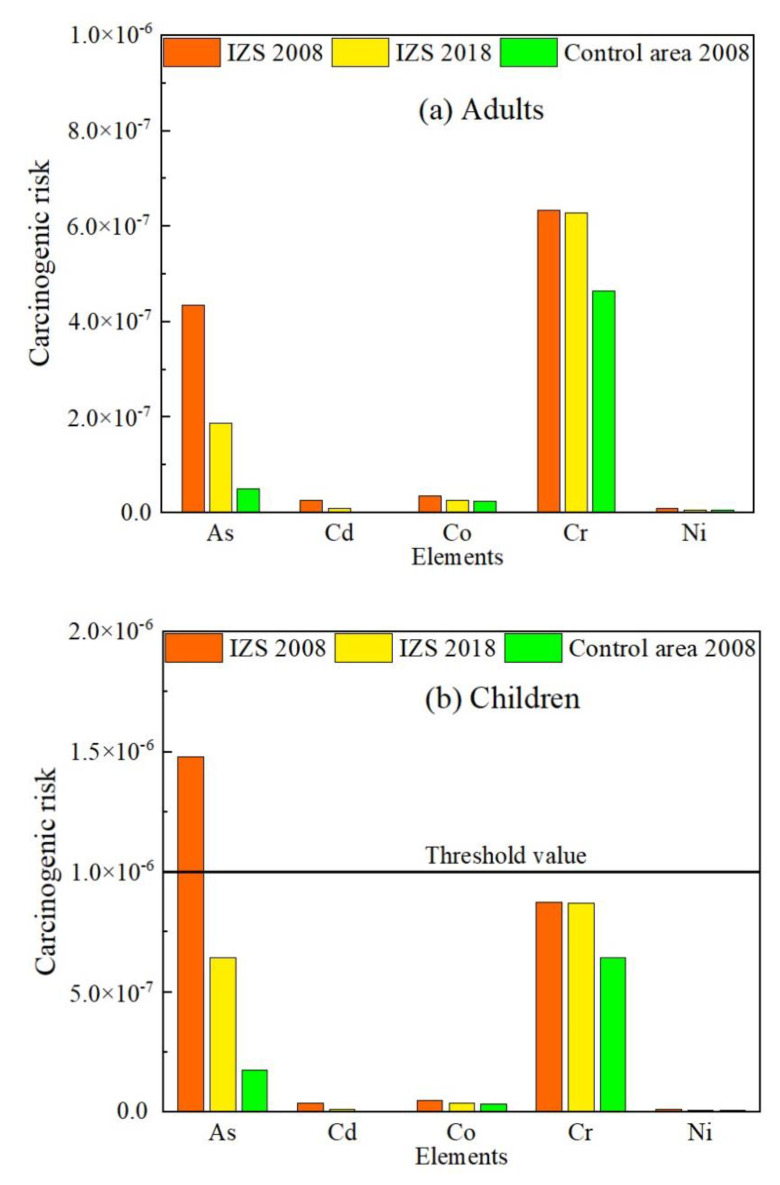
Carcinogenic risk of metal(loid)s in ground dust to adults (**a**) and children (**b**).

Figure 6 shows the carcinogenic risk of exposure to As, Cd, Co, Cr, and Ni in ground dust. The corresponding data is listed in Table S7. The risk decreased in the following order: As≈Cr>> Co> Cd> Ni. For adults, the calculated risk for all these five elements did not exceed threshold value (10^−6^–10^−4^) [55] in the IZS area both in 2008 and 2018. Although the levels of As and Cr for children were somewhat higher than those for adults, Cr was still in the acceptable value range. However, As represented the risk of 1.48 × 10^−6^ in 2008, indicating an unacceptable carcinogenic risk. For both subpopulations, the carcinogenic risk of Cd and Co exposure (10^−8^–10^−7^) fell below the threshold, and the risk of Ni exposure was within the lower range of 10^−8^–10^−9^. It suggested that Cd, Co, and Ni exposure pose a negligible risk. Although there is an obvious disparity in the results for the IZS and the control area, the carcinogenic risk of exposure to five metals in ground dust was generally low, with As for children as an exception.

## 4. Conclusions

In this study, the dynamics of fifteen heavy metal(loid)s in ground dust at a former IZS area in Guizhou were tracked over 2008–2018 after the cessation of IZS in 2006. Over half of the elements (e.g., Ag, As, Cd, Cu, Pb, Sb, Sn, Zn) decreased significantly over this period, thanks to the smoke reduction after the cessation of IZS activities and the treatment of smelting residues by blocking the source of heavy metal(loid)s, while the mean concentrations of Zn (1,287 mg/kg), Pb (762 mg/kg), Cd (7.76 mg/kg), and As (41.9 mg/kg) in 2018 was still high. Consequently, Pb and As represented the highest risk of non-carcinogenic adverse health impacts on the local residents, with the non-carcinogenic risk to local children in 2018 still higher than 1, suggesting that children were at high health risk. Therefore, continuous removal of remaining untreated smelting residues and potteries in the IZS area would be a top priority to further reduce the pollution of heavy metal(loid)s in ground dust. In addition, experiments with simulated gastric or lung fluids should be conducted in the future to have real data on potential bioaccesibility of these contaminants.

## Figures and Tables

**Table 1:** Concentrations of heavy metal(loid)s in ground dust of this study and the background values in agricultural soil in Guizhou and China (unit in mg/kg).

Elements	Range (Min-Max)			Arithmetic Mean (Mean ± SD)			Background Soils in Guizhou
	IZS Area 2008	IZS Area 2018	Control Area 2008	IZS Area 2008	IZS Area 2018	Control Area 2008	
Ag	0.37–7.98	0.47–4.25	0.23–0.36	2.80 ± 2.08	1.57 ± 1.13	0.31 ± 0.04	0.068
As	4.2–390.2	13.6–135.9	4.9–25.9	111.0 ± 96.0	41.9 ± 34.8	13.8 ± 5.2	14.89
Bi	0.31–12.29	0.63–20.80	0.47–1.17	3.66 ± 3.16	3.87 ± 5.39	0.70 ± 0.21	0.41
Cd	2.19–124.22	3.01–22.63	0.17–2.32	25.62 ± 21.93	7.76 ± 5.06	0.78 ± 0.61	0.313
Co	4–42	10–26	11–24	25 ± 9	18 ± 5	17 ± 3	17.38
Cr	32–195	55–179	53–109	104 ± 36	96 ± 33	76 ± 17	79.42
Cu	49–2442	35–216	65–3636	371 ± 413	97 ± 46	672 ± 1062	29.43
Hg	0.09–6.80	0.11–1.12	0.16–3.95	0.51 ± 1.08	0.32 ± 0.33	0.57 ± 0.95	0.103
In	0.04–2.78	0.10–2.28	0.06–0.14	0.60 ± 0.53	0.51 ± 0.57	0.09 ± 0.02	0.102
Ni	11–143	23–61	31–64	67 ± 32	44 ± 10	41 ± 10	32.84
Pb	220–6348	168–1974	40–163	2065 ± 1710	762 ± 647	78 ± 33	29.39
Sb	1.7–64.8	3.8–32.5	1.3–9.1	19.7 ± 14.3	10.9 ± 7.4	2.8 ± 2.0	1.35
Sn	1.98–126.48	3.10–25.64	2.61–6.90	20.37 ± 21.73	8.57 ± 5.46	3.98 ± 1.43	3.19
Tl	0.13–2.16	0.24–0.74	0.20–0.83	0.87 ± 0.44	0.42 ± 0.14	0.43 ± 0.13	0.712
Zn	256–8245	448–2848	123–569	3488 ± 2526	1287 ± 753	295 ± 149	89.94

Note: IZS area, artisanal zinc smelting area; Mean: arithmetic average; SD, standard deviation; Min, minimum; Max, maximum; Guizhou and China, background in surface soil (0–20 cm) of Guizhou (Soil Background Value in Guizhou Province) and China (National Background Value of Soil (Layer A)), respectively. Sample number for IZS area 2008, IZS area 2018 and control area 2008 is 37, 14, and 15, respectively.

**Table 2:** Comparison of mean values of heavy metal(loid)s in ground dust of different sites (unit in mg/kg).

Study Site	Ag	As	Bi	Cd	Co	Cr	Cu	Hg	In	Ni	Pb	Sb	Sn	Tl	Zn	Reference
IZS area 2008	2.80	111	3.66	25.6	25	104	371	0.51	0.60	67	2,065	19.7	20.37	0.87	3488	This study
IZS area 2018	1.57	41.9	3.87	7.76	18	96	97	0.32	0.51	44	762	10.9	8.57	0.42	1287	This study
Control area 2008	0.31	13.8	0.70	0.78	17	76	672	0.57	0.09	41	78	2.8	3.98	0.43	295	This study
Hezhang city, China	0.68	55.9	0.86	6.45	17.5	133	153	0.27	0.21	40.6	409	7.44	7.34	0.27	966	[46]
Zhuzhou city, China	2.49	89	12.3	41.4	13	125	139	0.92	4.06	40	956	15.8		1.08	2379	[41]
Huludao city, China				72.8			264	1.22			533				5271	[21]
Avilés city, Spain	3.76	37.6		45.1	10.9	109	370	2.93		43.3.	496	10.4			12,036	[45]
Guiyang city, China		11.28		0.62		131	130	0.33		61	67.8				186	[47]
Beijing city, China		4.88		0.59		92	83.1	0.16		32.5	60.9				281	[48]
Shanghai city, China				1.23		159	197			84.0	295				734	[49]
Hongkong, China				3.77			173				181				1450	[50]
Egypt		6.53		2.98		85.7	102			38.5	307		12.4		1839	[51]
Shiraz, Iran		6.58		0.5		67.2	136	1.05		77.52	116				403	[52]
Toronto, Canada				0.51		197.9	162			58.8	182.8				232.8	[53]

## Data Availability

Data is contained within the article and supplementary material.

## Supplementary Materials

The following are available online at https://www.mdpi.com/1660-4601/18/3/893/s1, Table S1 Exposure parameters for health risk assessment models; Table S2 Reference doses for non-carcinogenic and slope factors for carcinogenic metals; Table S3 Average *I_geo_* values and *I_geo_* classes of heavy metal(loid)s in ground dust; Table S4 Daily exposure amounts, hazard quotients, and hazard index of heavy metal(loid)s in ground dust to adults and children at the IZS area in 2008; Table S5 Daily exposure amounts, hazard quotients, and hazard index of heavy metal(loid)s in ground dust to adults and children at the IZS area in 2018; Table S6 Daily exposure amounts, hazard quotients, and hazard index of heavy metal(loid)s in ground dust to adults and children at the control area in 2008; Table S7 Cancer risk of metal(loid)s in ground dust to adults and children.
