# Supplementary material for: Heavy Metal(loid)s Contamination in Ground Dust and Associated Health Risks at a Former Indigenous Zinc Smelting Area
Source: Int J Environ Res Public Health. 2021 Jan 21;18(3):893. doi: 10.3390/ijerph18030893 (PMC7864351; doi:10.3390/ijerph18030893)
Supplement: Supplementary file 1 [file ijerph-18-00893-s001.pdf]

Supporting Information for

Heavy Metal(loid)s Contamination in Ground Dust and Associated Health Risks at a Former Indigenous Zinc Smelting Area

Shan Li [1,2], Xiangyang Bi [3], Zhonggen Li [1,4,*], Heng Wang [5], Xinyu Li [1,2], Xinbin Feng [1], Guangyi Sun [1,2], Ji Chen [1] and Bo Meng [1,*]

[1] State Key Laboratory of Environmental Geochemistry, Institute of Geochemistry, Chinese Academy of Sciences, Guiyang 550081, China; lishan@mail.gyig.ac.cn (S.L.); lixinyu@mail.gyig.ac.cn (X.L.); fengxinbin@mail.gyig.ac.cn (X.F.); sunguangyi@mail.gyig.ac.cn (G.S.); chenji@mail.gyig.ac.cn (J.C.)
[2] University of Chinese Academy of Sciences, Beijing 100049, China
[3] State Key Laboratory of Biogeology and Environmental Geology, School of Earth Sciences, China University of Geosciences, Wuhan 430074, China; bixy@cug.edu.cn
[4] School of Resources and Environment, Zunyi Normal College, Zunyi 563006, China
[5] School of Public Management, Guizhou University of Finance and Economics, Guiyang 550025, China; hengwang198510@126.com
* Correspondence: lizhonggencn@126.com (Z.L.), mengbo@mail.gyig.ac.cn (B.M.)

Table S1. Exposure parameters for health risk assessment models.

Symbol	Meanings	Unit	Parameter value		Reference
			Children	Adults	
C	Elements in street dust	mg·kg^{-1}	95% UCL*		This study
IngR	Ingestion rate	mg·day^{-1}	200	100	[1]
InhR	Inhalation rate	m^3·day^{-1}	7.6	20	[2]
EF	Exposure frequency	day·yr^{-1}	180		[3]
ED	Exposure duration	yr	6	24	[1,4]
BW	Average body weight	kg	15.9	56.8	[1,4,5]
AT	Average time	day	365×ED		[6]
SA	Skin area exposed	cm^2	1150	2145	[7]
SL	Skin adherence factor	mg·cm^{-2}·day^{-1}	0.2	0.07	[1,4]
ABS	Dermal absorption factor		0.03 for As;<br>0.001 for other elements		[8]
PEF	Particle emission factor	m^3·kg^{-1}	1.36×10^9		[1]
VF	Volatility factor	m^3 kg^{-1}	32,675.6		[1]
CR	Contact (absorption) rate		Ingestion: [CR = IngR]; Inhalation: [CR = InhR];<br>Dermal: [CR = SA × SL × ABS]		[1,4]

Note: 95% UCL means the upper limit of the 95% confidence interval for mean (UCL, upper confidence limit).

Table S2. Reference doses for non-carcinogenic and slope factors for carcinogenic metal(loid)s

Elements	RfD_{ing} [mg/(kg·day)]	RfD_{dermal} [mg/(kg·day)]	RfD_{inh} [mg/(kg·day)]	SF_{ing} (kg·day)/mg	SF_{dermal} (kg·day)/mg	SF_{inh} (kg·day)/mg	Reference
Ag	5.00×10^{-3}	9.00×10^{-4}	5.00×10^{-3}				[3,4,9]
As	3.00×10^{-4}	1.23×10^{-4}	3.00×10^{-4}	1.50	3.66	1.51×10^{1}	[3,10]
Cd	1.00×10^{-3}	1.00×10^{-5}	1.00×10^{-3}			6.30	[3,10]
Co	2.00×10^{-2}	1.60×10^{-2}	5.71×10^{-6}			9.80	[3,10]
Cr	3.00×10^{-3}	6.00×10^{-5}	2.86×10^{-5}			4.20×10^{1}	[3,10]
Cu	4.00×10^{-2}	1.20×10^{-2}	4.02×10^{-2}				[3,10]
Hg	3.00×10^{-4}	2.10×10^{-5}	8.57×10^{-5}				[3,10]
Ni	2.00×10^{-2}	5.40×10^{-3}	2.06×10^{-2}			8.40×10^{-1}	[3,10]
Pb	3.50×10^{-3}	5.25×10^{-4}	3.52×10^{-3}				[3,10]
Sb	4.00×10^{-4}	8.00×10^{-6}	4.00×10^{-4}				[3,10]
Zn	3.00×10^{-1}	6.00×10^{-2}	3.00×10^{-1}				[3,10]

Table S3. Average I_{geo} values and I_{geo} classes of heavy metal(loid)s in ground dust.

**Elements**	IZS area				Control area	
	2008 (No.=37)		2018 (No.=14)		2008 (No.=15)	
	I_{geo} value	I_{geo} class	I_{geo} value	I_{geo} class	I_{geo} value	I_{geo} class
Ag	4.38	5	3.64	4	1.6	2
As	1.72	2	0.57	1	-0.8	0
Bi	2.02	3	1.81	2	0.1	1
Cd	5.36	6	3.83	4	0.4	1
Co	-0.21	0	-0.61	0	-0.63	0
Cr	-0.27	0	-0.38	0	-0.68	0
Cu	2.55	3	1.00	1	2.6	3
Hg	1.04	2	0.50	1	1.2	2
In	1.48	2	-2.26	0	-0.8	0
Ni	0.28	1	-0.19	0	-0.28	0
Pb	4.99	5	3.60	4	0.7	1
Sb	2.88	3	2.19	3	0.2	1
Sn	1.59	2	0.65	1	-0.3	0
Tl	-0.47	0	-1.42	0	-1.4	0
Zn	4.19	5	3.00	3	0.9	1

Table S4. Daily exposure amounts, hazard quotients, and hazard index of heavy metal(loid)s in ground dust to adults and children at the IZS area in 2008.

Exposure indexes	Ag	As	Cd	Co	Cr	Cu	Hg	Ni	Pb	Sb	Zn
						Children					
ADD_{ing} (mg·kg⁻¹·day⁻¹)	2.24×10^{-5}	9.16×10^{-4}	2.11×10^{-4}	1.77×10^{-4}	7.46×10^{-4}	3.25×10^{-3}	5.56×10^{-6}	4.97×10^{-4}	1.69×10^{-2}	1.56×10^{-4}	2.77×10^{-2}
ADD_{inh} (mg·kg⁻¹·day⁻¹)	6.25×10^{-10}	2.56×10^{-8}	5.89×10^{-9}	4.95×10^{-9}	2.09×10^{-8}	9.09×10^{-8}	1.55×10^{-10}	1.39×10^{-8}	4.72×10^{-7}	4.37×10^{-9}	7.75×10^{-7}
ADD_{dermal}(mg·kg⁻¹·day⁻¹)	2.57×10^{-8}	3.16×10^{-5}	2.43×10^{-7}	2.04×10^{-7}	8.58×10^{-7}	3.74×10^{-6}	6.40×10^{-9}	5.72×10^{-7}	1.94×10^{-5}	1.80×10^{-7}	3.19×10^{-5}
ADD_{vapour}(mg·kg⁻¹·day⁻¹)							6.47×10^{-6}				
HQ_{ing}	4.48×10^{-3}	3.05	2.11×10^{-1}	8.86×10^{-3}	2.49×10^{-1}	8.14×10^{-2}	1.85×10^{-2}	2.49×10^{-2}	4.82	3.91×10^{-1}	9.24×10^{-2}
HQ_{inh}	1.25×10^{-7}	8.53×10^{-5}	5.89×10^{-6}	8.67×10^{-4}	7.29×10^{-4}	2.26×10^{-6}	5.36×10^{-7}	6.74×10^{-7}	1.34×10^{-4}	1.09×10^{-5}	2.58×10^{-6}
HQ_{dermal}	2.86×10^{-5}	2.57×10^{-1}	2.43×10^{-2}	1.27×10^{-5}	1.43×10^{-2}	3.12×10^{-4}	3.05×10^{-4}	1.06×10^{-4}	3.70×10^{-2}	2.25×10^{-2}	5.32×10^{-4}
HQ_{vapour}							7.55×10^{-2}				
HI	4.50×10^{-3}	3.31	2.35×10^{-1}	9.74×10^{-3}	2.64×10^{-1}	8.17×10^{-2}	9.43×10^{-2}	2.50×10^{-2}	4.86	4.14×10^{-1}	9.30×10^{-2}
						Adults					
ADD_{ing}(mg·kg⁻¹·day⁻¹)	3.08×10^{-6}	1.26×10^{-4}	2.90×10^{-5}	2.44×10^{-5}	1.03×10^{-4}	4.48×10^{-4}	7.65×10^{-7}	6.83×10^{-5}	2.32×10^{-3}	2.15×10^{-5}	3.81×10^{-3}
ADD_{inh}(mg·kg⁻¹·day⁻¹)	4.53×10^{-10}	1.85×10^{-8}	4.26×10^{-9}	3.58×10^{-9}	1.51×10^{-8}	6.58×10^{-8}	1.12×10^{-10}	1.01×10^{-8}	3.41×10^{-7}	3.16×10^{-9}	5.61×10^{-7}
ADD_{dermal}(mg·kg⁻¹·day⁻¹)	4.62×10^{-9}	5.67×10^{-6}	4.35×10^{-8}	3.66×10^{-8}	1.54×10^{-7}	6.72×10^{-7}	1.15×10^{-9}	1.03×10^{-7}	3.48×10^{-6}	3.23×10^{-8}	5.73×10^{-6}
ADD_{vapour}(mg·kg⁻¹·day⁻¹)							4.68×10^{-6}				
HQ_{ing}	6.15×10^{-4}	4.20×10^{-1}	2.90×10^{-2}	1.22×10^{-3}	3.42×10^{-2}	1.12×10^{-2}	2.55×10^{-3}	3.42×10^{-3}	6.63×10^{-1}	5.38×10^{-2}	1.27×10^{-2}
HQ_{inh}	9.05×10^{-8}	6.17×10^{-5}	4.26×10^{-6}	6.27×10^{-4}	5.28×10^{-4}	1.64×10^{-6}	3.88×10^{-7}	4.88×10^{-7}	9.69×10^{-5}	7.91×10^{-6}	1.87×10^{-6}
HQ_{dermal}	5.13×10^{-6}	4.61×10^{-2}	4.35×10^{-3}	2.29×10^{-6}	2.57×10^{-3}	5.60×10^{-5}	5.47×10^{-5}	1.90×10^{-5}	6.64×10^{-3}	4.04×10^{-3}	9.54×10^{-5}
HQ_{vapour}							5.46×10^{-2}				
HI	6.21×10^{-4}	4.66×10^{-1}	3.34×10^{-2}	1.85×10^{-3}	3.73×10^{-2}	1.12×10^{-2}	5.72×10^{-2}	3.44×10^{-3}	6.70×10^{-1}	5.78×10^{-2}	1.28×10^{-2}

Table S5. Daily exposure amounts, hazard quotients, and hazard index of heavy metal(loid)s in ground dust to adults and children at the IZS area in 2018.

Exposure indexes	Ag	As	Cd	Co	Cr	Cu	Hg	Ni	Pb	Sb	Zn
Children											
ADD_{ing} ($mg{\cdot}kg^{-1}{\cdot}day^{-1}$)	1.42×10^{-5}	3.97×10^{-4}	6.84×10^{-5}	1.34×10^{-4}	7.40×10^{-4}	7.92×10^{-4}	3.26×10^{-6}	3.22×10^{-4}	7.27×10^{-3}	9.71×10^{-5}	1.10×10^{-2}
ADD_{inh} ($mg{\cdot}kg^{-1}{\cdot}day^{-1}$)	3.98×10^{-10}	1.11×10^{-8}	1.91×10^{-9}	3.74×10^{-9}	2.07×10^{-8}	2.21×10^{-8}	9.11E-11	8.99×10^{-9}	2.03×10^{-7}	2.71×10^{-9}	3.08×10^{-7}
ADD_{dermal}($mg{\cdot}kg^{-1}{\cdot}day^{-1}$)	1.64×10^{-8}	1.37×10^{-5}	7.87×10^{-8}	1.54×10^{-7}	8.51×10^{-7}	9.11×10^{-7}	3.75×10^{-9}	3.70×10^{-7}	8.36×10^{-6}	1.12×10^{-7}	1.27×10^{-5}
ADD_{vapour}($mg{\cdot}kg^{-1}{\cdot}day^{-1}$)							3.79×10^{-6}				
HQ_{ing}	2.85×10^{-3}	1.32	6.84×10^{-2}	6.69×10^{-3}	2.47×10^{-1}	1.98×10^{-2}	1.09×10^{-2}	1.61×10^{-2}	2.08	2.43×10^{-1}	3.68×10^{-2}
HQ_{inh}	7.96×10^{-8}	3.70×10^{-5}	1.91×10^{-6}	6.54×10^{-4}	7.23×10^{-4}	5.50×10^{-7}	3.14×10^{-7}	4.36×10^{-7}	5.77×10^{-5}	6.78×10^{-6}	1.03×10^{-6}
HQ_{dermal}	1.82×10^{-5}	1.11×10^{-1}	7.87×10^{-3}	9.61×10^{-6}	1.42×10^{-2}	7.59×10^{-5}	1.78×10^{-4}	6.85×10^{-5}	1.59×10^{-2}	1.40×10^{-2}	2.11×10^{-4}
HQ_{vapour}							4.42×10^{-2}				
HI	2.87×10^{-3}	1.43	7.63×10^{-2}	7.35×10^{-3}	2.61×10^{-1}	1.99×10^{-2}	5.53×10^{-2}	1.61×10^{-2}	2.09	2.57×10^{-1}	3.70×10^{-2}
Adults											
ADD_{ing} ($mg{\cdot}kg^{-1}{\cdot}day^{-1}$)	1.96×10^{-6}	5.46×10^{-5}	9.41×10^{-6}	1.84×10^{-5}	1.02×10^{-4}	1.09×10^{-4}	4.48×10^{-7}	4.42×10^{-5}	1.00×10^{-3}	1.33×10^{-5}	1.52×10^{-3}
ADD_{inh}($mg{\cdot}kg^{-1}{\cdot}day^{-1}$)	2.88×10^{-10}	8.02×10^{-9}	1.38×10^{-9}	2.70×10^{-9}	1.50×10^{-8}	1.60×10^{-8}	6.59×10^{-11}	6.50×10^{-9}	1.47×10^{-7}	1.96×10^{-9}	2.23×10^{-7}
ADD_{dermal}($mg{\cdot}kg^{-1}{\cdot}day^{-1}$)	2.94×10^{-9}	2.46×10^{-6}	1.41×10^{-8}	2.76×10^{-8}	1.53×10^{-7}	1.64×10^{-7}	6.73×10^{-10}	6.64×10^{-8}	1.50×10^{-6}	2.00×10^{-8}	2.28×10^{-6}
ADD_{vapour}($mg{\cdot}kg^{-1}{\cdot}day^{-1}$)							2.74×10^{-6}				
HQ_{ing}	3.92×10^{-4}	1.82×10^{-1}	9.41×10^{-3}	9.19×10^{-4}	3.39×10^{-2}	2.72×10^{-3}	1.49×10^{-3}	2.21×10^{-3}	2.86×10^{-1}	3.34×10^{-2}	5.05×10^{-3}
HQ_{inh}	5.76×10^{-8}	2.67×10^{-5}	1.38×10^{-6}	4.74×10^{-4}	5.23×10^{-4}	3.98×10^{-7}	2.27×10^{-7}	3.16×10^{-7}	4.18×10^{-5}	4.91×10^{-6}	7.43×10^{-7}
HQ_{dermal}	3.27×10^{-6}	2.00×10^{-2}	1.41×10^{-3}	1.73×10^{-6}	2.54×10^{-3}	1.36×10^{-5}	3.20×10^{-5}	1.23×10^{-5}	2.86×10^{-3}	2.51×10^{-3}	3.79×10^{-5}
HQ_{vapour}							3.20×10^{-2}				
HI	3.95×10^{-4}	2.02×10^{-1}	1.08×10^{-2}	1.39×10^{-3}	3.70×10^{-2}	2.74×10^{-3}	3.35×10^{-2}	2.22×10^{-3}	2.89×10^{-1}	3.59×10^{-2}	5.09×10^{-3}

Table S6. Daily exposure amounts, hazard quotients, and hazard index of heavy metal(loid)s in ground dust to adults and children at the control area in 2008.

**Exposure indexes**	**Ag**	**As**	**Cd**	**Co**	**Cr**	**Cu**	**Hg**	**Ni**	**Pb**	**Sb**	**Zn**
Children											
ADD_{ing}($mg\cdot kg^{-1}\cdot day^{-1}$)	2.11×10^{-6}	1.07×10^{-4}	7.15×10^{-6}	1.23×10^{-4}	5.48×10^{-4}	8.07×10^{-3}	6.98×10^{-6}	3.01×10^{-4}	6.18×10^{-4}	2.52×10^{-5}	2.42×10^{-3}
ADD_{inh}($mg\cdot kg^{-1}\cdot day^{-1}$)	5.90E-11	2.98×10^{-9}	2.00×10^{-10}	3.43×10^{-9}	1.53×10^{-8}	2.26×10^{-7}	1.95E-10	8.40×10^{-9}	1.73×10^{-8}	7.03×10^{-10}	6.76×10^{-8}
ADD_{dermal}($mg\cdot kg^{-1}\cdot day^{-1}$)	2.43×10^{-9}	3.68×10^{-6}	8.22×10^{-9}	1.41×10^{-7}	6.30×10^{-7}	9.28×10^{-6}	8.03×10^{-9}	3.46×10^{-7}	7.10×10^{-7}	2.90×10^{-8}	2.78×10^{-6}
ADD_{vapour}($mg\cdot kg^{-1}\cdot day^{-1}$)							8.12×10^{-6}				
HQ_{ing}	4.22×10^{-4}	3.56×10^{-1}	7.15×10^{-3}	6.14×10^{-3}	1.83×10^{-1}	2.02×10^{-1}	2.33×10^{-2}	1.50×10^{-2}	1.76×10^{-1}	6.29×10^{-2}	8.07×10^{-3}
HQ_{inh}	1.18×10^{-8}	9.94×10^{-6}	2.00×10^{-7}	6.01×10^{-4}	5.35×10^{-4}	5.61×10^{-6}	6.73×10^{-7}	4.08×10^{-7}	4.90×10^{-6}	1.76×10^{-6}	2.25×10^{-7}
HQ_{dermal}	2.70×10^{-6}	2.99×10^{-2}	8.22×10^{-4}	8.82×10^{-6}	1.05×10^{-2}	7.74×10^{-4}	3.82×10^{-4}	6.40×10^{-5}	1.35×10^{-3}	3.62×10^{-3}	4.64×10^{-5}
HQ_{vapour}							9.48×10^{-2}				
HI	4.25×10^{-4}	3.86×10^{-1}	7.97×10^{-3}	6.75×10^{-3}	1.94×10^{-1}	2.03×10^{-1}	1.18×10^{-1}	1.51×10^{-2}	1.78×10^{-1}	6.66×10^{-2}	8.12×10^{-3}
Adults											
ADD_{ing}($mg\cdot kg^{-1}\cdot day^{-1}$)	2.90×10^{-7}	1.47×10^{-5}	9.83×10^{-7}	1.69×10^{-5}	7.54×10^{-5}	1.11×10^{-3}	9.60×10^{-7}	4.13×10^{-5}	8.49×10^{-5}	3.46×10^{-6}	3.33×10^{-4}
ADD_{inh}($mg\cdot kg^{-1}\cdot day^{-1}$)	4.27×10^{-11}	2.16×10^{-9}	1.45×10^{-10}	2.48×10^{-9}	1.11×10^{-8}	1.63×10^{-7}	1.41×10^{-10}	6.08×10^{-9}	1.25×10^{-8}	5.09×10^{-10}	4.90×10^{-8}
ADD_{dermal}($mg\cdot kg^{-1}\cdot day^{-1}$)	4.36×10^{-10}	6.61×10^{-7}	1.48×10^{-9}	2.53×10^{-8}	1.13×10^{-7}	1.67×10^{-6}	1.44×10^{-9}	6.20×10^{-8}	1.27×10^{-7}	5.20×10^{-9}	5.00×10^{-7}
ADD_{vapour}($mg\cdot kg^{-1}\cdot day^{-1}$)							5.88×10^{-6}				
HQ_{ing}	5.81×10^{-5}	4.89×10^{-2}	9.83×10^{-4}	8.44×10^{-4}	2.51×10^{-2}	2.78×10^{-2}	3.20×10^{-3}	2.07×10^{-3}	2.43×10^{-2}	8.65×10^{-3}	1.11×10^{-3}
HQ_{inh}	8.54×10^{-9}	7.19×10^{-6}	1.45×10^{-7}	4.35×10^{-4}	3.87×10^{-4}	4.06×10^{-6}	4.87×10^{-7}	2.95×10^{-7}	3.55×10^{-6}	1.27×10^{-6}	1.63×10^{-7}
HQ_{dermal}	4.85×10^{-7}	5.37×10^{-3}	1.48×10^{-4}	1.58×10^{-6}	1.89×10^{-3}	1.39×10^{-4}	6.87×10^{-5}	1.15×10^{-5}	2.43×10^{-4}	6.50×10^{-4}	8.33×10^{-6}
HQ_{vapour}							6.86×10^{-2}				
HI	5.86×10^{-5}	5.43×10^{-2}	1.13×10^{-3}	1.28×10^{-3}	2.74×10^{-2}	2.79×10^{-2}	7.19×10^{-2}	2.08×10^{-3}	2.45×10^{-2}	9.31×10^{-3}	1.12×10^{-3}

Table S7. Cancer risk of metal(loid)s in ground dust to adults and children.

Study area and period		As	Cd	Co	Cr	Ni
**Children**						
IZS	2008	1.48×10^{-6}	3.71×10^{-8}	4.85×10^{-8}	8.76×10^{-7}	1.17×10^{-8}
	2018	6.42×10^{-7}	1.20×10^{-8}	3.66×10^{-8}	8.68×10^{-7}	7.55×10^{-9}
Control area	2008	1.73×10^{-7}	1.26×10^{-9}	3.36×10^{-8}	6.43×10^{-7}	7.05×10^{-9}
Adults						
IZS	2008	4.34×10^{-7}	2.69×10^{-8}	3.51×10^{-8}	6.34×10^{-7}	8.44×10^{-9}
	2018	1.88×10^{-7}	8.72×10^{-9}	2.65×10^{-8}	6.28×10^{-7}	5.46×10^{-9}
Control area	2008	5.05×10^{-8}	9.11×10^{-10}	2.43×10^{-8}	4.65×10^{-7}	5.10×10^{-9}

Reference

1 USEPA. *Supplemental Guidance for Developing Soil Screening Levels for Superfund Sites*; OSWER 9355.4-24; Office of Solid Waste and Emergency Response:Washington, DC, USA, 2001.

2 Van den Berg, R. *Human Exposure to Soil Contamination: A Qualitative and Quantitative Analysis towards Proposals for Human Toxicological Intervention Values;* RIVM Report No. 725201011; National Institute of Public Health and Environmental Protection (RIVM): Bilthoven, The Netherlands, 1995.

3 Ferreira-Baptista, L. and De Miguel, E. Geochemistry and risk assessment of street dust in Luanda, Angola: A tropical urban environment. *Atmos Environ*, **2005**, *39*, 4501-4512.

4 USEPA. *Soil Screening Guidance: Technical Background Document*; EPA 540-R-95-128; U.S. Environmental Protection Agency: Washington, DC, USA, 1996.

5 Wang, X., Sato, T., Xing, B. and Tao, S. Health risks of heavy metals to the general public in Tianjin, China via consumption of vegetables and fish. *Sci Total Environ*, **2005**, *350*, 28-37.

6 USEPA. *Risk Assessment Guidance for Superfund, Volume I: Human Health Evaluation Manual*; EPA 540-1-89-002; U.S. Environmental Protection Agency: Washington, DC, USA, 1989.

7 Wang, Z.; Liu, S. Q.; Chen, X. M.; Lin, C. Y., Estimates of the exposed dermal surface area of Chinese in view of human health risk assessment. *Journal of Safety & Environment* **2008**, *8*, 152-156.(in Chinese)

8 USDOE. Available online: http://science.energy.gov/~/media/ budget/pdf/sc-budget-request-to-congress/fy-2004/Cong_Budget_2004_BER.pdf.

9 Sun, G.; Li, Z.; Liu, T.; Chen, J.; Wu, T.; Feng, X., Metal Exposure and Associated Health Risk to Human Beings by Street Dust in a Heavily Industrialized City of Hunan Province, Central China. *Int J Environ Res Public Health* **2017**, *14*, (3).

10 Wan, D.J.; Zhan, C.L.; Yang, G.L.; Liu, X.Q.; Yang, J.S. Preliminary assessment of health risks of potentially toxic elements in settled dust over Beijing urban area. *Int. J. Environ. Res. Public Health* **2016**, 13, 491.
